# Depression in Patients with Chronic Heart Failure: Correlation with Hemodynamic Parameters and Lipids’ Metabolism

**DOI:** 10.1192/j.eurpsy.2025.594

**Published:** 2025-08-26

**Authors:** S. Fedorov, A. K. Sikora

**Affiliations:** 1 Therapy and Family Medicine department of Post-graduate Faculty; 2 Psychiatry, Addictions and Medical Psychology Department, Ivano-Frankivsk National Medical University, Ivano-Frankivsk , Ukraine; 3 HOSPITAL OF THE MAZOWIECKIE VOIVODESHIP DREWNICA, Warsaw, Poland

## Abstract

**Introduction:**

Depression is a major concern in patients with chronic heart failure (CHF), with a prevalence of approximately 20-40%. It has been linked to worsened outcomes, including mortality and significant declines in physical and social functioning. Understanding the relationship between depression and hemodynamic as well as lipid metabolism parameters in CHF patients can provide insights into the underlying mechanisms of these worsened outcomes.

**Objectives:**

This study aims to evaluate the correlation between depression and central hemodynamic and lipid metabolism parameters in patients with chronic heart failure (CHF).

**Methods:**

The study involved 80 patients with CHF II-III NYHA classes caused by chronic coronary artery disease. They were divided into two groups: 20 without signs of depression and 60 with depression, as diagnosed using the Zung Self-Rating Depression Scale, Beck Depression Inventory, and Hamilton’s Depression Scale. Hemodynamic parameters were assessed using echocardiography (EchoCG), and lipid levels were measured in blood plasma. Statistical analyses were performed using t-tests, Mann–Whitney U tests, and correlation coefficients to determine relationships between variables.

**Results:**

Depressive symptoms were prevalent in 71.6% of the CHF patients, with severe depression observed in 3.3%. Significant correlations were found between depression severity and blood pressure, triglyceride levels (r = 0.7, p < 0.05), and key echocardiographic measures such as left atrium diameter (r = 0.57, p < 0.05), end-diastolic size (r = 0.53, p < 0.05), and ejection fraction (r = -0.29, p < 0.05). Additionally, situational anxiety was significantly correlated with serum urea levels (r = 0.42, p < 0.05), triglycerides (r = 0.91, p < 0.05), and echocardiographic parameters.

**Image 1:**

**Figure:**
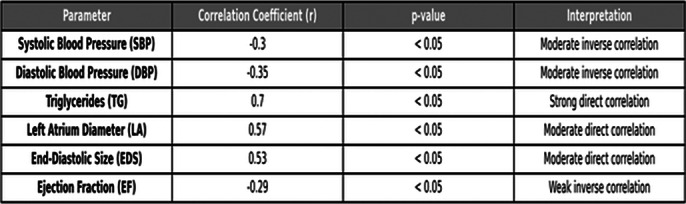

**Image 2:**

**Figure:**
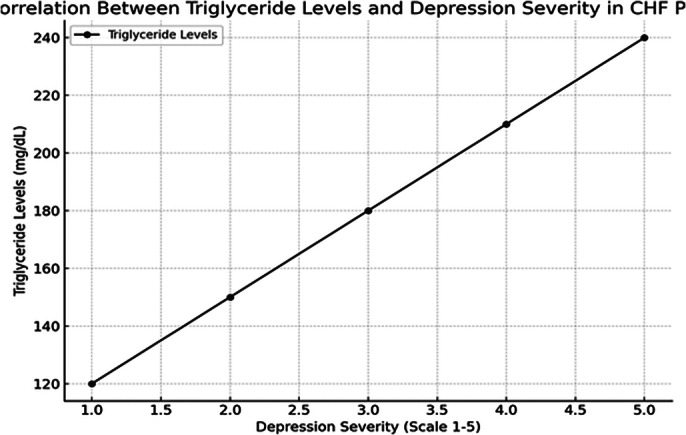

**Conclusions:**

Patients with CHF and depression exhibit more severe central hemodynamic and lipid metabolism disorders than those without depression. These findings suggest that addressing depression in CHF management may mitigate some of the adverse effects on cardiovascular health.

**Disclosure of Interest:**

None Declared

